# University Departments of Rural Health and cancer research in Aboriginal and Torres Strait Islander populations

**DOI:** 10.1016/j.lanwpc.2025.101621

**Published:** 2025-06-29

**Authors:** Samantha Bay, Emma V. Taylor, Charmaine Green, Sandra C. Thompson

**Affiliations:** Western Australian Centre for Rural Health, University of Western Australia, 167 Fitzgerald Street, Geraldton, Western Australia 6530, Australia

Cancer is one of the most common causes of illness and death in Australia, and it has been well documented that people living in rural and remote areas have poorer outcomes.^1^ A large proportion of Aboriginal and Torres Strait Islander (henceforth respectfully referred to as Indigenous) people live in regional, rural and remote locations. Cancer is the leading cause of death for Indigenous Australians, who are more likely to have poor outcomes compared to non-Indigenous Australians regardless of rurality.^1^

University Departments of Rural Health (UDRHs) were first established more than 25 years ago as part of a strategy to build students’ interest in health careers in rural areas and strengthen the rural health workforce. UDRHs are in a unique position to engage regional and rural communities and to improve health research, services and outcomes of rural residents and Indigenous Australian people. Staff employed by UDRHs have engaged in research for many years, and considerable research contributions to rural health knowledge have been made because of UDRH staff scholarship despite underinvestment in rural research.^2^ UDRH Indigenous staff have also been internationally recognised for their contributions to cancer research.^3^

An analysis of Indigenous health research from UDRHs published between 2010 and 2021 has been previously reported.^4^ Using the same source, we examined publications between 2010 and 2023 relating to cancer research to comment on the contributions of UDRHs to Indigenous cancer research. The methods and results of this search are described in Supplementary file S1, and Supplementary file S2 summarises the number of cancer publications which UDRHs contributed to between 2010 and 2023. Of 248 cancer research publications, 61 (24·6%) were related to Indigenous Australians and cancer. Unsurprisingly, the number of published cancer articles increased over time, and articles with a focus on Indigenous Australians increased as a proportion of cancer research. Most publications were research articles and were co-authored with researchers from other organisations.

Indigenous cancer research conducted by UDRHs has highlighted the importance of capacity building of healthcare providers and Indigenous communities. Focus areas include health literacy, culturally safe care, palliative care, and navigating the multifaceted challenges in cancer care. For example, one study showed the importance of educating healthcare workers about the needs and priorities of Indigenous Australian people in end-of-life care to improve staff confidence in providing care to Indigenous patients and providing culturally safe care.^5^ UDRH publications also described building the capacity of the regional and rural workforce, including Indigenous healthcare workers. Of note, one study showed increased confidence in engaging in conversations about end-of-life care, facilitating relationships and collaboration with palliative care services after building knowledge and skills of Indigenous healthcare workers about palliative care delivery models.^6^ Studies have identified important enablers of culturally safe service provision (such as provision of psychological support, meeting patient and cultural needs, practical assistance, and advocating for Indigenous health),^7^^,^^8^ and factors that contribute to continuity of care for Indigenous Australians with cancer (including timely communication and information exchange, collaborative approaches, streamlined processes, flexible care delivery, and patient-centred care and support).^9^ Capacity building requires a sustained commitment to funding and development of research skills of both Indigenous Australians and rural researchers and will also bring benefits from two-way learning.^10^

There are ongoing challenges associated with conducting cancer research in rural settings and with Indigenous Australians. Factors include acquiring funding and costs of running programs in rural locations, engagement of participants, the training and availability of a suitable workforce both for cancer care and for research. UDRHs have made efforts to overcome some of these challenges, for example, using technology and Australian Rural Health Education Network (ARHEN) linkages to collaborate with researchers and health education networks around Australia. Over the years, UDRHs have built strong partnerships with local and Indigenous communities which enable research projects and cancer health promotion initiatives to be conducted in rural areas and in collaboration with Indigenous peoples and services.

The contributions of UDRHs to cancer research extend beyond peer reviewed publications as there has been development of resources and approaches in regional and rural areas to support Indigenous people in their cancer journey. An example of this is the ‘Whisper No More’ training program for healthcare staff. This resource built on previous research as captured in a community report ‘A Whispered Sort of Stuff’^11^ which summarised Aboriginal people’s beliefs about cancer and their experiences of cancer care in Western Australia and involved UDRH authors and rural Indigenous Australians. UDRH research has contributed to informing Indigenous Australian cancer policy, for example, in critically examining bowel cancer screening,^12^, ^13^, ^14^ primary health care settings,^9^^,^^15^ lung cancer,^16^ and organisational policies.^17^

## Recommendations

Indigenous staff members at UDRHs play pivotal roles in research, including into major causes of Indigenous morbidity and mortality such as cancer. The current National Aboriginal and Torres Strait Islander Health Plan highlights the importance of implementing projects that are co-designed, led by Indigenous people, and which respect Indigenous data sovereignty.^18^ Indigenous peoples’ lived experiences are invaluable to the development of projects that will significantly improve cancer outcomes and services for Indigenous Australians. Indigenous researchers can draw on their personal and their communities’ understanding of underlying cultural, social, economic and psychological determinants that contribute to poorer cancer outcomes, to implement projects that overcome barriers to cancer diagnosis and care. Sustained funding and support for Indigenous Australian UDRH staff is essential for continuing their key roles in improving Indigenous cancer outcomes, sustaining interest and engagement of local communities, providing invaluable insights to rural health issues and solutions, ensuring co-design of ethical cancer research, and building capacity within local rural communities and the health workforce.

Many publications captured in ARHEN’s database were by Higher Degree by Research (HDR) students, with significant projects that have contributed to capacity building of rural and Indigenous healthcare workers and improving healthcare for Indigenous people with cancer. Universities and the Australian Government can continue to support improving rural cancer outcomes by increasing the funding and support for Indigenous and non-Indigenous HDR students conducting research projects or research translation activities in rural areas.

Most research in Indigenous populations is descriptive in nature.^4^ Researchers should consider the potential for research fatigue on Indigenous Australians, and research must aim to improve outcomes of Indigenous communities in a sustainable way. Projects must progress within timeframes that enable translation and implementation of findings. The requirements for human research ethics assessment are often duplicative, onerous and expensive, particularly when the research crosses the jurisdiction of multiple committees.^19^ While rigorous ethics assessments are essential to ensure safety, resources for research must allow time for capacity building and research translation,^20^ and not be impeded by multiple ethics applications at the front end.

Rural researchers at UDRHs were often partners in the implementation of projects rather than leaders, as many UDRHs do not have the “research machine” infrastructure of research institutes and must allocate their limited resources wisely. Successful interventions for regions with small population numbers will likely benefit from more collaborative approaches with and across rural regions, requiring new approaches to optimising research for rural Indigenous cancer. Funding local staff and rural projects allows for resources to flow to and remain in the regions. This will enable UDRHs to continue their work in advancing cancer care and improving cancer outcomes for Indigenous Australians, with ongoing projects enabled by supportive stakeholders, funders and partnerships with Indigenous communities.

### Conclusions

UDRHs have contributed to cancer research through bringing both rural and Indigenous lenses to research efforts in improving cancer outcomes. UDRHs’ contributions to Indigenous related cancer research and initiatives are evident in multiple domains: exploring ways to improve service provision, particularly cultural safety in service delivery; attention on screening and palliative care; amplifying the voices of Indigenous cancer patients, Indigenous families and health professionals; partnering and bridging the gaps between academia, health services and Indigenous communities; and building capacity in Indigenous staff, Indigenous communities, and rural healthcare providers. Over and above the research itself, UDRHs have provided support to Indigenous researchers gaining research skills, increasing community members’ understanding of health, and through training the next generation of health professionals. These contributions reflect the sustained government investment in UDRHs as rural academic centres, employment of committed rurally based Indigenous and non-Indigenous staff, utilising close relationships to rural residents, and links with wider health, academic and policy networks.

## Contributors

The authors collectively have over 40 years of research in rural contexts and working with UDRHs.

**Cultural backgrounds of authors**

The authorship team consisted of Indigenous Australian and non-Indigenous researchers. CG is a Wajarri, Badimaya and Wilunyu woman of the Yamaji Nation from Western Australia. The non-Indigenous team members collectively have decades of experience in Indigenous health research.

Conceptualization: SB, EVT, CG, SCT.

Formal analysis: EVT.

Project administration: SB, SCT.

Visualization: EVT.

Writing—original draft: SB, SCT.

Writing—review & editing: SB, EVT, CG, SCT.

## Data sharing statement

Not applicable, there is no primary data for this paper. Supplementary files S1 and S2 summarises UDRH cancer publications.

## Declaration of interests

SCT is a member of the Aboriginal Advisory Committee of the Cancer Council of Western Australia, however this membership was not associated with this manuscript. All other authors declare no conflicts of interest.
