# Supplementary material for: University Departments of Rural Health and cancer research in Aboriginal and Torres Strait Islander populations
Source: Lancet Reg Health West Pac. 2025 Jun 29;61:101621. doi: 10.1016/j.lanwpc.2025.101621 (PMC12414349; doi:10.1016/j.lanwpc.2025.101621)
Supplement: Supplementary file S1 [file mmc1.docx]

**Supplementary file 1.**

**Methods and results: UDRH cancer research 2010-2023**

**Methods**

As part of their efforts to improve rural health, rural health services and Indigenous health, 19 UDRHs from all states and territories in Australia (except the Australian Capital Territory) share knowledge and strategies through the Australian Rural Health Education Network (ARHEN). ARHEN has established and maintains a database of peer-reviewed articles published since 2010 which have at least one UDRH author. This collection allows reflection of the role that UDRHs have in research and to consider the importance of those contributions in particular areas. Here, we comment on the contributions of UDRHs towards cancer research, particularly in Indigenous Australian populations.

For this article, authors conducted a search of cancer research in ARHEN’s database by searching: title OR abstract OR keywords that contained “cancer” and repeating the search with the keyword “palliative”. Publications between 2010 – 2023 were included in the search. The articles relating to Indigenous Australians were identified by further searching: “Aboriginal” OR “Indigenous” OR “Torres Strait” OR “First Nations” using the title, keyword and abstract fields.

Data about the co-authorship affiliations, nature of the studies, key findings, and implications for future research were extracted by authors after reading the full texts of the publications. Authors also searched Google Scholar and recorded the number of citations associated with each publication as of 27^th^ February 2025.

**Results**

A total of 248 cancer research publications were identified in ARHEN’s database, and 61 (24·6%) were related to Indigenous Australians and cancer. Unsurprisingly, the number of cancer articles increased over time, and articles with a focus on Indigenous Australians increased as a proportion of cancer research from 20% (2010-2016) to 27% (2017-2023). The majority (n=51; 83.6%) were research articles, 9 were review articles and 1 was a commentary. Only 3 papers were produced solely by UDRH researchers, with the majority co-authored with researchers from other organisations: Industry partner(s) in collaboration with UDRHs (n=5; 8.2%), metropolitan-based university departments in collaboration with UDRHs (n=22; 36.1%), and multiple organisations in collaboration with UDRHs (n=31; 50.8%). The table below displays a list and a summary of key findings of publications from ARHEN’s database relating to cancer in Indigenous Australians published between 2010-2023, and Supplementary file 2 summarises the number of cancer publications from AHREN’s database about Indigenous Australians between 2010-2023.

Given UDRHs’ interest in nursing and allied health training and support, UDRH research in cancer often extended beyond treating cancer and the physical health problems associated with cancer. The cancer research relating to Indigenous Australians covered a wide range of topics, with commonly explored areas being service delivery (n=18; 29.5%), staff perspectives (n=14; 23.0%), patient perspectives (n=10; 16.4%) and end-of-life/palliative care (n=10; 16.4%). Other topics included screening, traditional beliefs and medicines, continuity of care and peer-support for patients. The wide breadth of publications shows that UDRHs have been making a significant contribution to Indigenous cancer research, with some papers cited more than 100 times (Christou & Thompson, 2012; Shahid et al., 2011).

**Summary of publications (2010-2023) with UDRH contributor(s) about cancer in Aboriginal and Torres Strait Islander people**

| Author(s) & year | Title | Google Scholar Citations | Topics | Key findings | Implications and future research |
| --- | --- | --- | --- | --- | --- |
| (Banham et al., 2023) | The effect of general practice contact on cancer stage at diagnosis in Aboriginal and non-Aboriginal residents of New South Wales | 0 | Service delivery | Older Indigenous Australians diagnosed with cancer were likely to have more comorbidities and greater socioeconomic disadvantage than other Australians, which may increase the likelihood of being diagnosed at a more advanced stage.  More frequent GP contact was positively associated earlier diagnosis of cancer. | More research is needed to investigate:  The characteristics of GP contacts that are related to earlier stage at diagnosis.  Different effects of GP contact by primary site.  How the severity of comorbidity conditions’ impact on quality of life and health preferences. |
| (Bernardes et al., 2019) | Unmet supportive care needs among Indigenous cancer patients across Australia | 27 | Needs | Most Indigenous Australians living with cancer experience unmet supportive care needs. Physical/psychological and practical/cultural concerns were identified as priority areas for Indigenous cancer patients. Moderate to high unmet needs most occurred in physical/psychological (46%) and practical/cultural (34%) domains. 'Money worries' was the most frequently reported unmet need (20%). | Interventions needed to target physical, psychological, practical and cultural needs. |
| (Carey et al., 2016) | Improving the quality of life of palliative and chronic disease patients and carers in remote Australia with the establishment of a day respite facility | 18 | Palliative care;  Respite | The establishment of a respite service in a remote town improved case management and care coordination of chronic and complex patients as well as improved medication compliance and symptom management. | The importance of community-based, culturally sensitive respite services in improving the quality of life for palliative and chronic disease patients and their carers in remote areas. |
| (Chan et al., 2023) | Radiation Therapy and Indigenous Peoples in Canada and Australia: Building Paths Toward Reconciliation in Cancer Care Delivery | 8 | Service delivery | Progress has been made in increasing access to radiotherapy for Indigenous peoples in Canada and Australia.  Cancer care delivery can be strengthened through recruiting Indigenous staff, education, partnerships, and research. | Increasing Indigenous cultural training among health professionals, recruiting Indigenous staff and medical trainees to oncology, and strengthening partnerships between oncology providers and Indigenous community for research and systems change is vital. |
| (Christou et al., 2010) | Australia's national bowel cancer screening program: Does it work for Indigenous Australians? | 65 | Screening | The NBCSP is not reaching many Indigenous Australians in the target group. Factors contributing to lower participation include how participants are selected, the way the screening kit is distributed, the nature of the test and comprehensiveness of its contents, cultural perceptions of cancer and prevailing low levels of knowledge and awareness of bowel cancer and the importance of screening. | Modify the NBCSP to be more accessible and tailored to the needs of Indigenous Australians and other minority groups, through a more community-based and primary care-integrated approach. |
| (Christou & Thompson, 2012) | Colorectal cancer screening knowledge, attitudes and behavioural intention among Indigenous Western Australians | 149 | Screening | Awareness and knowledge of colorectal cancer (CRC) screening were low, although both were significantly associated with exposure to media advertising.  Nearly two-thirds (63%) of respondents intended to participate in screening, however 84% said they would participate on a doctor’s recommendation.  Predictors of screening intention included: perceived self-efficacy in completing the FOBT test, participating in any cancer screening in the past two years and greater knowledge about bowel cancer. | Address the barriers created by the current design of the NBCSP, such as the postal distribution of the FOBT and requirement for self-screening.  Develop health promotion and education strategies that focus on improving knowledge, awareness, and self-efficacy around CRC screening among Indigenous Australians |
| (Christou & Thompson, 2013) | Missed opportunities in educating Aboriginal Australians about bowel cancer screening: whose job is it anyway? | 24 | Education resources | Despite positive feedback on the flipchart's design, it was underutilized. Only one-third of respondents had used it.  Reasons for underutilization included the perception that Indigenous health education should be handled by Indigenous health professionals, health staff not prioritising bowel cancer education and lack of opportunity or time. | Greater recognition by all health professionals of their potential role in Indigenous health education.  Need for awareness, time, and specific training for effective tool implementation. |
| (Cuesta-Briand et al., 2015) | Addressing unresolved tensions to build effective partnerships: lessons from an Aboriginal cancer support network | 12 | Peer-support for patients | Unresolved tensions between Indigenous and non-Indigenous participants regarding the need for structure versus flexibility in the operations of an Indigenous cancer support network negatively impacted the working relationship and threatened the network's sustainability.  Indigenous participants preferred a flexible, organic approach, while mainstream service providers believed a more structured approach was needed for sustainability. | Address and resolve tensions between different perspectives and world views in order to build strong, effective partnerships between service providers and Indigenous communities. |
| (Cuesta-Briand et al., 2016) | 'Connecting tracks': exploring the roles of an Aboriginal women's cancer support network | 12 | Peer-support for patients | The main roles of the Indigenous women's cancer support network were:  Linking and connecting people and services, acting as a "cultural broker" to provide a culturally safe space for Indigenous women to engage with cancer services and health promotion/screening initiatives.  Providing emotional support, though there was tension between Indigenous stakeholders valuing cultural appropriateness and MSPs prioritizing clinical safety.  Engaging in grassroots health promotion and education initiatives to address the "silence about cancer" in the Indigenous community.  Providing practical and financial support. | Ensure adequate ongoing funding and resources to support the sustainability of culturally appropriate Indigenous-led initiatives.  Build collaborative relationships between Indigenous organisations and mainstream cancer service providers  Value and honour Indigenous approaches and expertise. |
| (Darcey et al., 2019) | The association between mammographic density and breast cancer risk in Western Australian Aboriginal women | 4 | Screening;  Pathology | Mammographic density is a strong predictor of breast cancer risk in Indigenous women in WA, similar to its role in non-Indigenous women.  Despite having generally lower mammographic density than non-Indigenous women, Indigenous women with higher density were found to have a greater risk of breast cancer compared to their peers with lower density. | Breast cancer risk prediction models and efforts to standardize risk-associated mammographic density measures should take Indigenous status into account.  Indigenous women with dense breasts could potentially benefit from supplemental screening using a modality other than mammography. |
| (Davidson et al., 2013) | The experience of lung cancer in Aboriginal and Torres Strait Islander peoples and what it means for policy, service planning and delivery | 24 | Patient perspectives | High smoking rates, cultural beliefs, geographic remoteness, socioeconomic disadvantages, and systemic racism create substantial barriers to accessing timely and effective lung cancer care.  Many Indigenous Australians experience late-stage diagnoses and receive less aggressive treatment than non-Indigenous Australians, further impacted by limited specialist services in rural areas, financial burdens, and family obligations that often hinder relocation for treatment.  Cultural beliefs, such as perceptions of cancer as a "death sentence" or punishment, alongside mistrust in healthcare institutions due to historical discrimination, also deter early intervention and adherence to treatment. | Improve data collection and identification of Indigenous populations to better understand the lung cancer burden.  Adopt a national policy approach to address lung cancer disparities, with Indigenous ownership, participation and control.  Implement culturally appropriate smoking cessation programs and health literacy strategies.  Address health disparities through coordinated policy initiatives across health and social policy, with effective engagement and partnership with Indigenous communities |
| (de Witt et al., 2017) | Identification of Australian Aboriginal and Torres Strait Islander Cancer Patients in the Primary Health Care Setting | 10 | Identifying Indigenous status | There was no standardized way to identify Indigenous cancer patients across primary health care (PHC) clinics.  Health service information systems, search functions and capacities of systems, and staff skill in extracting data using electronic Patient Care Information Systems (PCIS) varied between clinics. | Improving the identification processes and ensuring consistent data management practices across PHC clinics could enhance the monitoring and evaluation of cancer care for Indigenous patients.  It is also important for PHC staff to receive training and support to utilize PCISs efficiently and effectively. |
| (de Witt et al., 2018) | "It's Just Presence," the Contributions of Aboriginal and Torres Strait Islander Health Professionals in Cancer Care in Queensland | 12 | Staff perspectives;  Follow-up care | Indigenous health professionals are key enablers to support the needs of Indigenous patients when accessing cancer care.  Culturally safe care, practical assistance, psychological support, determination of patient needs, and advocacy for Indigenous health were identified as enablers to support the needs of Indigenous cancer patients. | Health professionals need to work together and be skilled in the delivery of culturally competent care to improve outcomes for Indigenous people with cancer.  Organizational policies and practices are crucial to support health professionals to provide culturally competent and responsive cancer care. |
| (de Witt et al., 2020) | Communication, Collaboration and Care Coordination: The Three-Point Guide to Cancer Care Provision for Aboriginal and Torres Strait Islander Australians | 19 | Staff perspectives;  Follow-up care | The following five themes are crucial to improve the continuity and coordination of cancer care between the PHC service and the treating hospital for Indigenous cancer patients: timely communication and information exchange; collaborative approaches; streamlined processes; flexible care delivery; and patient-centred care and support. | Improving communication, collaboration and care coordination between services is vital to the provision of quality cancer care for Indigenous Australians.  Health policy and funding must be designed to incorporate these aspects across services and settings as a strategy to improve cancer outcomes for Indigenous Australians. |
| (de Witt et al., 2021) | Aboriginal and Torres Strait Islander patients' cancer care pathways in Queensland: Insights from health professionals | 4 | Staff perspectives;  Follow-up care | The provision of culturally competent care, effective communication, coordination and collaboration between services along the entire cancer pathway are vital to enhance care continuity for Indigenous Australians. | Implementing the recommendations outlined in the Optimal Care Pathway for Aboriginal and Torres Strait Islander people with cancer framework may help improve cancer care continuity for Indigenous patients with cancer. |
| (Diaz et al., 2019) | Service Level Factors Associated with Cervical Screening in Aboriginal and Torres Strait Islander Primary Health Care Centres in Australia | 10 | Screening;  Service delivery | Substantial variation in cervical screening rates among 135 Indigenous Primary Health Care (PHC) centres.  Service-level factors, including longer participation in continuous quality improvement (CQI) programs and centres located in very remote areas, were linked to higher screening coverage. | Strategies for improving the delivery of cervical screening by Indigenous PHC centres should be devised by, and for, Aboriginal and Torres Strait Islander women.  To maximize the benefit of CQI programs, it is necessary to understand how organizational, environmental and system-level factors moderate the relationship between CQI participation and service delivery. |
| (Gall, Anderson, Adams, et al., 2019) | An exploration of healthcare providers' experiences and perspectives of Traditional and complementary medicine usage and disclosure by Indigenous cancer patients | 32 | Staff perspectives; Traditional medicine | Health professionals’ perceptions of traditional and complementary medicines (T&CM) were grouped into six themes: concern about risk; no 'real' benefits; perception of T&CM and conventional medicine as antithetical; barriers to disclosure; 'patients' choice' a double-edged sword; and providers' lack of knowledge about T&CM.  Most health professionals lacked knowledge of T&CM and had concerns around negative interactions with conventional treatments. | Health professionals require greater understanding of T&CM to inform discussion with Indigenous cancer patients, and to ensure patient care is not compromised.  It is important to foster health care interactions where Indigenous patients feel comfortable to discuss T&CM usage. |
| (Gall, Anderson, Diaz, et al., 2019) | Exploring traditional and complementary medicine use by Indigenous Australian women undergoing gynaecological cancer investigations | 7 | Patient perspectives; Traditional medicine | 86% of Indigenous women undergoing clinical investigations for gynaecological cancer were using traditional and complementary medicines (T&CM).  Participants reported major challenges in communicating with healthcare providers about T&CM, due to lack of trust and rapport. | Strategies to facilitate culturally appropriate doctor-patient communication around T&CM are urgently needed to foster trust and transparency in gynaecological cancer care for Indigenous women. |
| (Gall et al., 2018) | Traditional and Complementary Medicine Use Among Indigenous Cancer Patients in Australia, Canada, New Zealand, and the United States: A Systematic Review | 53 | Traditional medicine | Usage of traditional and complementary medicines (T&CM) by Indigenous cancer patients in Australia, Canada, New Zealand, and the United States varied.  T&CM were mostly used concurrently with conventional cancer treatments to meet their spiritual, emotional, social, and cultural needs of Indigenous cancer patients. However, in a minority of cases, bush, traditional, and herbal medicines were used as an alternative to conventional treatments. | Additional research is required to explore the use and disclosure of T&CM among Indigenous cancer patients to help ensure safe, coordinated care for Indigenous cancer patients with shared decision making and improved communication. |
| (Haigh et al., 2016) | Talking about the not talked about: use of, and reactions to, a DVD promoting bowel cancer screening to Aboriginal people | 9 | Screening; Health promotion | The DVD developed to promote bowel cancer screening among Indigenous Australians was well-received and considered a suitable educational resource. However, the DVD was not widely distributed or utilized, like a previous flipchart resource.  The underutilization of the DVD was attributed to the lack of engagement of health service providers with the National Bowel Cancer Screening Program. | Improve distribution and follow-up strategies for health promotion resources like the DVD.  Increase involvement of primary care services in the distribution and follow-up of FOBT kits for the NBCSP.  Provide training and clearly articulate the role of health service providers, especially nurses, in promoting the NBCSP |
| (Lethborg et al., 2022) | Culturally Informed, Codesigned, Supportive Care for Aboriginal and Torres Strait Islander People With Cancer and Their Families | 15 | Service delivery | Hospital staff and Indigenous organisations worked together to develop, implement, and evaluate a culturally informed model of supportive cancer care for Indigenous people and their families.  The new evidence-based model of care incorporated culturally informed identification of needs, culturally appropriate engagement, and maintenance of respectful connections to develop codesigned care plans with tailored information and provide practical support and advocacy. | Supportive care needs of Indigenous people living with cancer are often complex and multifaceted, but strengths are links with community and culture.  Social workers can help to improve access and promote a culturally responsive model of care.  Appropriate support needs to be codesigned with the patient, their family, Indigenous Hospital Liaison Officers, and community. |
| (Lyford et al., 2018) | An Exploration of Underrepresentation of Aboriginal Cancer Patients Attending a Regional Radiotherapy Service in Western Australia | 16 | Service delivery | Indigenous cancer patients who received treatment at the regional radiotherapy service were satisfied with the care and support they received, despite the complex challenges they faced in accessing and continuing treatment.  Service providers suggested several reasons for the low numbers of Indigenous patients presenting at the radiotherapy service, including late cancer diagnosis, perceptions of cultural insensitivity, financial barriers, and issues with accurately recording patients' Indigenous status. | Provide a supportive environment that addresses the access challenges faced by rural Indigenous cancer patients from initial diagnosis through to follow-up care.  Educate Indigenous people about cancer symptoms and the importance of seeking timely medical advice. |
| (Martini et al., 2011) | Colorectal cancer screening in rural and remote areas: analysis of the National Bowel Cancer Screening Program data for South Australia | 33 | Screening | Indigenous participation in the National Bowel Cancer Screening Program in South Australia was 0.5%.  Bowel cancer screening participation was lower for older rural and remote residents, men, Indigenous people, lower socioeconomic groups and those living in the Far North subdivision of SA. | Older rural and remote residents, men, Indigenous peoples and those living in Far North SA need specifically targeted bowel cancer screening services. More research is required to understand the barriers faced by these population groups. |
| (McLean et al., 2019) | The distribution and determinants of mammographic density measures in Western Australian Aboriginal women | 4 | Screening; pathology | Indigenous women exhibited lower absolute dense area (DA) and percent dense area (PDA) compared to non-Indigenous women, indicating that they have, on average, less mammographic density.  The lower mammographic density in Indigenous women may improve the sensitivity of mammographic screening. | This study suggests that mammographic screening is a particularly good test for Indigenous Australian women.  Further research is needed to explore the underlying reasons for the lower mammographic density in Indigenous women and to investigate the relationship between mammographic density measures and breast cancer risk. |
| (Meiklejohn et al., 2018) | Community-identified recommendations to enhance cancer survivorship for Aboriginal and Torres Strait Islander people | 15 | Patient perspectives; Service delivery | Key action areas and recommendations to enhance cancer survivorship were identified: establishing a community cancer advocate and peer support program; availability and use of a cancer-specific Indigenous primary healthcare worker and hospital-based Indigenous patient navigator; as well as adoption of question prompt lists and cancer survivorship care plans. | Increased support and commitment to providing patient-centred follow-up cancer care across health services and by funding bodies is needed to ensure health system change. |
| (Meiklejohn et al., 2017) | Follow-up cancer care: perspectives of Aboriginal and Torres Strait Islander cancer survivors | 35 | Patient perspectives; Follow-up care | Study highlighted the need of timely and informative discharge information, strong therapeutic relationships between patients and tertiary and primary health professionals and provision of responsive and flexible follow-up care | The importance of strong therapeutic relationships between patients and tertiary and primary health professionals.  Provision of discharge summaries or care plans at discharge is important for survivors and general practitioners.  Alternative means for follow-up could be investigated for rural survivors to facilitate convenient and cost-effective follow-up care. |
| (Olver et al., 2021) | Communicating cancer and its treatment to Australian Aboriginal and Torres Strait Islander patients with cancer: a qualitative study | 8 | Staff perspectives; Service delivery | Six themes were identified: create a safe environment, engender trust and build rapport; employ specific communication strategies to explain cancer; obtain support from Aboriginal and Torres Strait Islander staff; consider culture which involves collective decision making, strong connection to country and community; anticipate conflicts between Western medicine and Indigenous culture; develop personal qualities of good communicators. | Create a teaching tool for health students, based on these learnings, then testing it to see how well it equips workers to communicate effectively with Aboriginal and Torres Strait Islander patients with cancer. |
| (Panozzo et al., 2023) | Bridging cultures in palliative care: A qualitative study of the care of Indigenous Australians with advanced illness | 5 | Staff perspectives; Palliative care | Four themes were identified regarding provision of palliative care for Indigenous patients: the intersection of cultures – Indigenous peoples, health and palliative care; the importance of Indigenous Hospital Liaison Officers (IHLOs) in bridging the cultural divide; health professionals devolving responsibility of providing culturally appropriate care to IHLOs; the need for a more holistic, culturally aware provision of palliative care. | System level change is required to ensure early involvement of IHLOs in Indigenous patient palliative care, improved cultural understanding through cultural safety training and health policy that supports the IHLO role. |
| (Pilkington et al., 2017) | Perspectives of Aboriginal women on participation in mammographic screening: a step towards improving services | 31 | Patient perspectives; Screening | Indigenous women in the study were generally willing to have a mammogram.  Motivating factors were having a genetic predisposition, a desire to maintain their health and set an example.  Key barriers to screening participation included lack of understanding about the screening process, inadequacies in cultural appropriateness in the screening program, cultural beliefs about cancer, and competing life demands.  Enablers to screening participation included culturally appropriate education and support from other Indigenous women in the community. | The higher participation rates for Indigenous women in Western Australia demonstrate the success of the strategies put in place by BreastScreen WA.  Existing efforts should be supported and barriers to screening participation identified by this study must be addressed. |
| (De Plaza et al., 2023) | Health System Enablers and Barriers to Continuity of Care for First Nations Peoples Living with Chronic Disease | 2 | Barriers to care; Continuity of care | Barriers to continuity of care for First Nations people with chronic disease included a lack of community initiatives, health and social care networks, and coaching and peer support.  Enablers of continuity of care included care adapted to patients’ cultural beliefs and behavioural, personal, and family influences; continued and trusting relationships among providers, patients, and caregivers; and provision of flexible, consistent, adaptable care along the continuum. | Sustainable continuity of care strategies must be co-designed and well-funded. Strategies should be flexible and include coaching and peer support across the lifespan. |
| (Ristevski et al., 2020) | Understanding Aboriginal Peoples' Cultural and Family Connections Can Help Inform the Development of Culturally Appropriate Cancer Survivorship Models of Care | 49 | Community perspectives; | Culture and family were central to treatment and survivorship experiences of Indigenous patients with cancer.  Gaps in information sharing and communication occurred between health professionals and patients and their families. Cancer had a financial impact on Indigenous patients and their families, with access to services depending on family support. | Understanding the importance of Indigenous peoples' cultural and family connections can help to inform the development of culturally safe cancer survivorship models of care. |
| (Shahid, Bessarab, et al., 2013) | Improving palliative care outcomes for Aboriginal Australians: service providers' perspectives | 39 | Staff perspectives; Palliative care | Palliative care provider participants reported a lack of understanding of Indigenous culture and being uncertain of the needs and priorities of Indigenous people during end-of-life care. Some participants believed that few Indigenous people understood palliative care.  Managing needs for non-medical support due to socioeconomic disadvantage and dealing with crises and conflicts over funeral arrangements caused tensions between Indigenous patients and service providers. | Early referral to palliative care is important for Indigenous patients in demonstrating and maintaining a caring therapeutic relationship. Paramount to meeting the needs for Indigenous patients was access to appropriate information and logistical, psychological and emotional support.  Need to employ an Indigenous worker in the palliative care team as well as increased cultural safety training for care providers. |
| (Shahid et al., 2010) | "If you don't believe it, it won't help you": use of bush medicine in treating cancer among Aboriginal people in Western Australia | 85 | Community perspectives; Traditional medicine | Some Indigenous Australians use traditional bush medicine and healing practices as part of their cancer treatment, as it helps them connect with their cultural heritage and spirituality.  Spiritual beliefs and a holistic view of health are important factors that influence some Indigenous patients' choices in cancer treatment, including the use of traditional medicine. | Health service providers should recognize and understand the use of traditional Indigenous medicine and healing, as this can improve Indigenous people's access and engagement with mainstream health services.  Further research is needed to understand the therapeutic value of traditional Indigenous medicine, so that it can be better integrated with Western medical approaches. |
| (Shahid, Durey, et al., 2013) | Identifying barriers and improving communication between cancer service providers and Aboriginal patients and their families: the perspective of service providers | 91 | Staff perspectives; Service delivery | Cancer Service Providers' lack of knowledge about the needs of Indigenous people with cancer and Indigenous patients' limited understanding of the Western medical system were major barriers to communication.  Communication was impeded by language differences, differing communication styles, and differing concepts of time, which created challenges for timely appointments and adherence to treatment plans. | Ensure cultural competency at the clinical, organizational, and system level by recruiting and training Indigenous staff, providing cultural safety training to all staff, and improving patient record systems to better identify Indigenous patients. |
| (Shahid, Ekberg, et al., 2018) | Experiential learning to increase palliative care competence among the Indigenous workforce: an Australian experience | 17 | Workforce capacity building | Supervised clinical placements with specialist palliative care services increased Indigenous health practitioners’ confidence in engaging in conversations about end of life (EOL) care and supported ongoing collaboration with palliative care services.  Management support was critical and placements with more experience in caring for Indigenous people were preferred.  Better engagement and outcomes were observed in programs that included Indigenous staffing and leadership.  Effective preplacement and postplacement preparation and mentoring were crucial. | Areas for program improvement included enhancing postplacement follow-up activities to ensure sustained skill development and support for the participants. |
| (Shahid et al., 2011) | 'Nowhere to room ... nobody told them': logistical and cultural impediments to Aboriginal peoples' participation in cancer treatment | 159 | Community perspectives; | A need for practical and emotional support in all aspects of the cancer journey was identified, particular for rural and remote patients.  Infrastructure and logistical problems that impeded Indigenous patients' access to cancer treatment included transport, accommodation, travel and service expenses, concerns about the hospital environment, displacement from family and lack of appropriate support persons. | System-level changes are needed to ensure cultural safety, social support, and better coordination between cancer treatment services and primary healthcare to improve outcomes for Indigenous people with cancer. |
| (Shahid, Taylor, et al., 2018) | Key features of palliative care service delivery to Indigenous peoples in Australia, New Zealand, Canada and the United States: a comprehensive review | 85 | Service delivery | Commonly expressed preferences of Indigenous people regarding palliative care: to die close to or at home; involvement of family and community; and the integration of cultural practices.  Barriers to palliative care included inaccessibility, affordability, lack of awareness of services, perceptions of palliative care, and culturally inappropriate services.  Strategies to address these gaps included: community engagement and ownership; flexibility in approach; continuing education and training; a whole-of-service approach; and local partnerships among multiple agencies. | Key features of culturally safe palliative care include flexibility, adaptability to the context, engaging local communities and family involvement in decision-making.  Additional research is required to explore the palliative care needs and experiences of Indigenous people living in urban areas. |
| (Shahid et al., 2016) | Factors contributing to delayed diagnosis of cancer among Aboriginal people in Australia: a qualitative study | 66 | Community perspectives; Staff perspectives; Service delivery | Multiple factors at the contextual, health system, and patient levels contribute to delayed cancer diagnosis among Indigenous Australians.  Contextual factors included the intergenerational impact of colonization, racism, and socioeconomic deprivation, which negatively affected trust in healthcare professionals.  Health service-related factors included limited access to medical services, long waiting periods for appointments and diagnostic tests, and a shortage of culturally competent health professionals.  Patient appraisal of symptoms and decision-making were significant, as many patients experienced fear, denial, and embarrassment regarding their symptoms, leading to procrastination in seeking help. | Focus on the primary care sector and encourage GPs to be more proactive in investigating potential cancer symptoms and facilitating timely referrals.  Implement community-based cancer awareness programs to educate Indigenous people about the importance of early diagnosis and treatment, aiming to dispel fears associated with cancer.  Use patient navigators and telehealth services to address barriers related to remoteness and lack of access to healthcare services. |
| (Shepherdson et al., 2022) | Young-Onset Gastrointestinal Adenocarcinoma Incidence and Survival Trends in the Northern Territory, Australia, with Emphasis on Indigenous Peoples | 8 | Incidence; Mortality | There is increased incidence of young-onset gastrointestinal (GI) adenocarcinomas in the NT for all residents aged 18-50 years.  Indigenous patients have lower incidence but worse survival across all GI subsites. | More research and targeted, culturally safe Indigenous community-focussed programs are needed for early detection and patient-centred management of GI adenocarcinomas. |
| (Smith, 2012) | A long way from home: Access to cancer care for rural Australians | 45 | Barriers to care | Rural Australians have significantly higher cancer mortality rates compared to their urban counterparts.  The main challenge faced by rural Australians in accessing cancer care is geographic isolation and the need to travel long distances.  Other barriers include financial strain, disruption to family life and the lack of specialist medical and allied health services in rural areas.  Rural Indigenous patients face additional barriers and experience even higher cancer mortality rates compared to the general rural population. | Government investment in regional cancer care infrastructure is essential.  Staffing the new facilities requires innovative workforce solutions including managed care pathways, outreach programs, models of shared care, and the use of telemedicine.  Models of cancer care for Indigenous people must address issues of cultural safety. |
| (Spelten et al., 2021) | Palliative Care in Rural Aboriginal Communities: Conversations Around Experiences and Needs | 7 | Community perspectives; Palliative care | There is a lack of understanding among rural Indigenous community members about palliative care and limited awareness of available palliative care services, which affects service utilization.  There is a need for clear information about palliative care services and practical supports available.  Concerns were raised about limited cultural awareness among healthcare providers.  The home-based nature of palliative care was not a barrier provided that appropriate respect for cultural practices was displayed. | Addressing cultural needs and improving understanding and awareness are necessary to overcome barriers to accessing palliative care for Indigenous people living in rural areas. |
| (Taylor, Haigh, Shahid, Garvey, Cunningham, Holloway, et al., 2018) | Australian cancer services: a survey of providers’ efforts to meet the needs of Indigenous patients | 6 | Staff perspectives; Service delivery | Indigenous cancer patients represented a small proportion of the overall patient load in most participating centres, with less than 5% of total patients being Indigenous in most cases.  Almost half (47%) of the centres reported seeing more than 10 Indigenous patients a year.  The most common initiatives reported were having links with Indigenous health organisations (74%), making a dedicated effort to address the needs of Indigenous patients (69%), and creating partnerships with Indigenous communities (69%).  Fewer respondents (58%) indicated that they had established specific programs or services for Indigenous patients and their families, and only 55% had policies guiding interactions with Indigenous patients. | Information from this study can assist cancer service providers to identify gaps in current services, plan new service delivery initiatives and ultimately improve Indigenous cancer outcomes. |
| (Taylor, Haigh, Shahid, Garvey, Cunningham, & Thompson, 2018) | Cancer Services and Their Initiatives to Improve the Care of Indigenous Australians | 25 | Staff perspectives; Service delivery | Many Indigenous-specific programs and initiatives are being implemented by cancer services across Australia to provide culturally appropriate care, but details of these initiatives are not widely known.  Key activities included employing Indigenous staff, providing cultural awareness training, incorporating flexible clinical practices, and making changes to the physical environment to ensure cultural safety.  Challenges included a lack of awareness among service providers regarding what other services were doing and limited opportunities for information sharing, particularly in regional and remote areas. | Need for enhanced collaboration, networking, and partnerships among cancer services, particularly between Indigenous and mainstream health service providers, to share successful strategies and initiatives.  Further research to evaluate these programs and initiatives and showcase the more effective approaches to Indigenous cancer care. |
| (Taylor et al., 2021) | "The support has been brilliant": experiences of Aboriginal and Torres Strait Islander patients attending two high performing cancer services | 17 | Patient perspectives; Staff perspectives; Service delivery | Three experiences were shared by most Indigenous cancer patients and family members interviewed in this study: a positive experience while receiving treatment at the two cancer services; challenges navigating the health system in the time between first experiencing systems and reaching the cancer service; and the importance of family support, while acknowledging the burden on family and carers. | Health services must make an ongoing commitment to strengthen the cultural safety of their services.  Need for more comprehensive coordination between primary and tertiary care providers.  Further research is needed to identify and evaluate successful cancer service delivery initiatives for Indigenous Australians. |
| (Taylor et al., 2020) | "We're very much part of the team here": A culture of respect for Indigenous health workforce transforms Indigenous health care | 49 | Staff perspectives; Patient perspectives; Service delivery | Eight themes were identified from the way that the two services supported their Indigenous workforce: strong executive leadership, a proactive employment strategy, the Indigenous Health Unit, the Indigenous Liaison Officer, multidisciplinary team inclusion, professional development, work environment and a culture of respect.  Positive outcomes from having a strong Indigenous workforce included: improved patient outcomes, increased patient engagement, better adherence to treatment, increased recruitment and retention of Indigenous staff and improved job satisfaction. | Positive patient outcomes and a strong Indigenous health workforce can be achieved when a health service has committed leadership with Indigenous health as a core value, commits to an inclusive and enabling culture, and develops specific support structures appropriate for Indigenous staff. |
| (Taylor, Lyford, et al., 2022) | Putting Policy into Practice: How Three Cancer Services Perform against Indigenous Health and Cancer Frameworks | 6 | Staff perspectives; Patient perspectives; Service delivery | Three Australian cancer services were compared to two national best practice guidelines.  While two services performed well against the National Safety and Quality Health Service (NSQHS) User Guide for Aboriginal and Torres Strait Islander Health (User Guide), all three struggled with the Education, Prevention, Screening and Early Diagnosis elements of the National Aboriginal and Torres Strait Islander Cancer Framework (Cancer Framework).  The importance of a whole-of-organisation approach when addressing and embedding the six actions of the User Guide.  Health services which have successfully implemented the User Guide are in a stronger position to implement the Cancer Framework and Cancer Pathway. | The treatment-focused Optimal Care Pathway for Aboriginal and Torres Strait Islander People with Cancer may be a more appropriate framework for tertiary services.  More research is needed to identify and evaluate successful cancer service delivery programs for Indigenous Australians. |
| (Taylor, Thackrah, et al., 2022) | Improving Access to Cancer Treatment Services in Australia's Northern Territory-History and Progress | 2 | Service delivery | The Northern Territory (NT) has succeeded in advocating for cancer patients and their families by increasing the cancer care infrastructure and services, allowing patients to stay closer to home and Country, improving access to specialist expertise and improving cancer outcomes. | Increased travel assistance and support and appropriate accommodation is needed for cancer patients.  Research on effective strategies to improve the recruitment and retention of health professionals working in cancer care, particularly Indigenous health professionals and health professionals working in remote settings.  Research into the effectiveness and appropriateness of telehealth as a means of delivering cancer care and treatment in remote settings and to identify issues specific to the Territory. |
| (Thackrah et al., 2022) | Perspectives of Aboriginal People Affected by Cancer on the Need for an Aboriginal Navigator in Cancer Treatment and Support: A Qualitative Study | 2 | Patient perspectives; Barriers to care; Continuity of care | Patients and carers identified many gaps in cancer service delivery and numerous challenges, including a lack of stable accommodation upon relocation, the financial burden related to time away and accessing treatment, being “off-Country”, and miscommunication with health professionals.  All participants supported the creation of an Indigenous Patient Navigator role to address shortfalls in cancer service delivery, especially for patients from rural and remote communities. | Evaluation of the new role would be required to assess the Indigenous Patient Navigator’s contribution to improved cancer care and outcomes for Indigenous patients. |
| (Thompson et al., 2019) | Passing on wisdom: exploring the end-of-life wishes of Aboriginal people from the Midwest of Western Australia | 15 | End of life | Indigenous people were willing to engage in discussions about end-of-life wishes when provided with a safe and culturally appropriate environment.  Key issues included concerns about wills, preferences regarding burial versus cremation, and the high costs associated with funerals.  Cancer patients emphasized the importance of family involvement in end-of-life decision-making and highlighted the potential for family conflict if wishes were not clearly communicated. | Providing opportunities for Indigenous people to discuss grief and loss around end-of-life issues can be beneficial.  Resources like the Palliative Care Australia's Dying to Talk materials and sorting cards can be useful to facilitate discussions about end-of-life preferences among Indigenous people. |
| (Thompson et al., 2011) | Not just bricks and mortar: planning hospital cancer services for Aboriginal people | 59 | Service delivery; Treatment environments | Five themes describing concerns about the hospital environment emerged: being alone and lost in a large system; poor communication; practical issues such as costs, transportation, and family responsibilities; importance of Indigenous support persons; and need for connection to community.  While the design of the hospital environment was important, it was secondary to the necessity of building trust and fostering culturally safe interactions. | Promotion of cultural safety, support for Indigenous family and respecting the importance of place and community to Indigenous patients are crucial in improving cancer outcomes.  Recommendations for the physical design of health services included an Indigenous Welcome Desk; welcoming physical environment; large hospital rooms to accommodate extended Indigenous families. |
| (Thompson et al., 2014) | Making progress: the role of cancer councils in Australia in indigenous cancer control | 59 | Policy; Institutional supports; Interventions | Since the 2006 review most Cancer Councils had increased activities aimed at improving Indigenous cancer outcomes, although Indigenous staff numbers remained low, and no Cancer Council had an Indigenous Board member.  There was evidence of enhanced partnerships with Indigenous organizations and increased acknowledgment of Indigenous issues in policy documents.  Some Councils had developed culturally relevant resources and training programs.  Challenges such as sustaining relationships, resource limitations, and the need for greater cultural competence persisted. | Cancer Councils should continue to recruit and support Indigenous staff through peer mentorship programs and ensure staff cultural competence through training.  Develop Indigenous-specific action plans, strategic engagement with Indigenous communities, and increased representation on Boards and committees. |
| (Tranberg et al., 2016) | Factors influencing cancer treatment decision-making by indigenous peoples: a systematic review | 35 | Community perspectives; Decision making | Socio-economic and cultural factors that had the potential to influence cancer treatment decision-making among Indigenous people were grouped into four themes: Spiritual beliefs, encompassing fatalistic views about cancer as a death sentence; Cultural influences, emphasizing the importance of family and community in decision-making and the shame associated with cancer diagnoses; Communication, highlighting the barriers Indigenous individuals face in discussing cancer with healthcare professionals and family members; and Existing healthcare systems and structures, which reflect the challenges of accessing appropriate care, particularly for those in rural or remote areas. | Further research is needed on investigating cancer treatment decision-making among Indigenous peoples, health professionals' knowledge of and attitudes towards Indigenous people's spirituality and ‘best practice’ in health professional communication with Indigenous people. |
| (Valery et al., 2020b) | Patterns of primary health care service use of Indigenous Australians diagnosed with cancer | 10 | Service use | Indigenous Australians diagnosed with cancer frequently utilize primary health care (PHC) services.  Frequency of PHC visits was higher among patients living in remote areas and those from socioeconomically disadvantaged backgrounds.  Higher rates of PHC visits were also observed in male patients, patients with greater comorbidity, those who did not undergo surgery, and those receiving chemotherapy and/or radiotherapy. | PHC services and general practitioners (GPs) play a critical role in the cancer care continuum for Indigenous Australians, particularly in remote and disadvantaged communities.  Remote PHC services require appropriate resourcing and support to provide these services. |
| (Valery et al., 2020a) | Are general practitioners getting the information they need from hospitals and specialists to provide quality cancer care for Indigenous Australians? | 0 | Continuity of care | There are significant deficits in the communication between hospitals/specialists and primary health care (PHC) services. 36.5% of audited patient records lacked a discharge summary and 40.0% of patients visited their PHC service before a discharge summary was available.  While most discharge summaries contained important information about the patient’s cancer, 42.4% did not include details about the discharge medication regimen. | The lack of timely and comprehensive discharge summaries can adversely affect the quality of cancer care provided by GPs, which may lead to delays in follow-up care, mismanagement of medications, and poorer health outcomes for Indigenous cancer patients.  These gaps should be addressed through better standardization of discharge summaries, ensuring they include all necessary information, and implementing systems to ensure timely transfer of these summaries to GPs |
| (van Schaik & Thompson, 2012) | Indigenous beliefs about biomedical and bush medicine treatment efficacy for indigenous cancer patients: a review of the literature | 28 | Patient perspectives; Western medicine; Traditional medicine | Indigenous Australian beliefs about the efficacy of cancer treatments were grouped into five themes: concerns regarding the toxicity and side effects of biomedical treatments; a disconnect between Indigenous patients and HPs often resulting in miscommunication and mistrust; fears related to the need for relocation to urban hospitals for treatment; differing beliefs about disease aetiology; and biomedical treatments failing to address holistic health. | Actively involve Indigenous patients in the design and process of their care.  Consider the practical challenges faced by Indigenous patients, such as transportation and separation from family, when developing treatment plans. |
| (Ward et al., 2011) | Equity of colorectal cancer screening: cross-sectional analysis of National Bowel Cancer Screening Program data for South Australia | 44 | Screening | Participation in the National Bowel Cancer Screening Program was lower for men, socially disadvantaged groups, and people from metropolitan and remote areas of South Australia. There were also inequities in participation based on Indigenous status and language spoken at home. | Geographic areas and population groups with lower participation may benefit from targeted approaches to screening, including public education campaigns and interventions by health professionals. |
| (Woods & Johnson, 2018) | Delay in commencement of palliative care service episodes provided to Indigenous and non-Indigenous patients: cross-sectional analysis of an Australian multi-jurisdictional dataset | 10 | Palliative care | The timeliness benchmark was met for both Indigenous and non-Indigenous patients. Indigenous Australians are slightly more likely to experience delays between being designated ready for care and commencing the episode of care. | Although the timeliness benchmark is being met for Indigenous Australians in palliative care, they may experience delays, particularly if younger, and especially at first encounter with a service.  Qualitative research is required to explore determinants of delay in initiating palliative care episodes. |
| (Woods, Johnson, et al., 2021) | Collaborative data familiarisation and quality assessment: Reflections from use of a national dataset to investigate palliative care for Indigenous Australians | 6 | Palliative care; data quality assessment | There was a noticeable improvement in the completeness of patient records from participating specialist palliative care services over the study period.  The data were error-free with respect to many credibility and consistency checks, with anomalies detected reported to data managers. | External researchers performing data quality assessment can play an important role in identifying anomalies and providing feedback to data managers, which can contribute to the improvement of data curation processes. |
| (Woods et al., 2020) | Symptom-Related Distress among Indigenous Australians in Specialist End-of-Life Care: Findings from the Multi-Jurisdictional Palliative Care Outcomes Collaboration Data | 9 | Palliative care | First-episode frequencies of symptom-related distress were similar for both Indigenous and non-Indigenous patients in both inpatient and community palliative care settings.  In final pre-death assessments both groups exhibited similar occurrences of moderate-to-severe distress when care was provided in hospital settings.  In community settings, Indigenous patients had a lower risk of moderate-to-severe distress from overall symptom occurrence compared to non-Indigenous patients. | The findings suggest a reasonable equivalence of end-of-life outcomes for Indigenous patients who have been accepted for specialist palliative care, indicating that the care provided is relatively equitable in terms of managing symptom-related distress. |
| (Woods, Katzenellenbogen, et al., 2021) | Occurrence and timely management of problems requiring prompt intervention among Indigenous compared with non-Indigenous Australian palliative care patients: a multijurisdictional cohort study | 2 | Palliative care; service delivery | The frequency and duration of unstable phases (indicating unanticipated deterioration in a patient’s condition) were similar between Indigenous and non-Indigenous patients when receiving specialist palliative care.  The benchmark of resolving 90% of unstable phases within three days was not met for either Indigenous or non-Indigenous patients. | There is no clear evidence of greater occurrence or prolongation of unanticipated deterioration among Indigenous patients accessing specialist palliative care services in hospital or the community. |
| (Woods et al., 2019) | Indigenous compared with non-Indigenous Australian patients at entry to specialist palliative care: Cross-sectional findings from a multi-jurisdictional dataset | 10 | Palliative care; service use | Indigenous Australians, especially those residing outside major cities, were substantially underrepresented in specialist palliative care services.  Indigenous palliative care patients were younger, a higher proportion were female, and a larger proportion resided outside major cities.  The clinical status at entry to palliative care was similar between Indigenous and non-Indigenous patients, indicating that those who do access care do not face greater clinical challenges at the outset | Improved linkage of data across palliative care services and between palliative care data and other health datasets would facilitate longitudinal investigation of care provided to patients with life-limiting illnesses throughout their illness journeys and may improve data quality including accuracy of identification of Indigenous patients. |

**References**

Banham, D., Roder, D., Thompson, S., Williamson, A., Bray, F., & Currow, D. (2023). The effect of general practice contact on cancer stage at diagnosis in Aboriginal and non-Aboriginal residents of New South Wales. *Cancer Causes & Control*, *34*(10), 909-926.

Bernardes, C. M., Diaz, A., Valery, P. C., Sabesan, S., Baxi, S., Aoun, S., Thompson, S. C., & Lashbrook, M. (2019). Unmet supportive care needs among Indigenous cancer patients across Australia. *Rural Remote Health*, *19*(3). <https://doi.org/10.22605/RRH4660>

Carey, T. A., Schouten, K., Wakerman, J., Humphreys, J. S., Miegel, F., Murphy, S., & Arundell, M. (2016). Improving the quality of life of palliative and chronic disease patients and carers in remote Australia with the establishment of a day respite facility. *BMC Palliat Care*, *15*(1), 62. <https://doi.org/10.1186/s12904-016-0136-1>

Chan, J., Griffiths, K., Turner, A., Tobias, J., Clarmont, W., Delaney, G., Hutton, J., Olson, R., Penniment, M., & Bourque, J.-M. (2023). Radiation therapy and indigenous peoples in Canada and Australia: building paths toward reconciliation in cancer care delivery. *International Journal of Radiation Oncology* Biology* Physics*, *116*(2), 421-429.

Christou, A., Katzenellenbogen, J. M., & Thompson, S. C. (2010). Australia's national bowel cancer screening program: Does it work for Indigenous Australians? *BMC Public Health*, *10*(10), 373. <http://www.biomedcentral.com/1471-2458/10/373>

Christou, A., & Thompson, S. C. (2012). Colorectal cancer screening knowledge, attitudes and behavioural intention among Indigenous Western Australians. *BMC Public Health*, *12*, 528. <https://doi.org/10.1186/1471-2458-12-528>

Christou, A., & Thompson, S. C. (2013). Missed opportunities in educating Aboriginal Australians about bowel cancer screening: whose job is it anyway? *Contemp Nurse*, *46*(1), 4084-4111. <https://doi.org/10.5172/conu.2013.4084>

Cuesta-Briand, B., Bessarab, D., Shahid, S., & Thompson, S. C. (2015). Addressing unresolved tensions to build effective partnerships: lessons from an Aboriginal cancer support network. *Int J Equity Health*, *14*(1), 122. <https://doi.org/10.1186/s12939-015-0259-7>

Cuesta-Briand, B., Bessarab, D., Shahid, S., & Thompson, S. C. (2016). 'Connecting tracks': exploring the roles of an Aboriginal women's cancer support network. *Health Soc Care Community*, *24*(6), 779-788. <https://doi.org/10.1111/hsc.12261>

Darcey, E., Lloyd, R., Cadby, G., Pilkington, L., Redfern, A., Thompson, S. C., Saunders, C., Wylie, E., & Stone, J. (2019). The association between mammographic density and breast cancer risk in Western Australian Aboriginal women [journal article]. *Breast Cancer Research and Treatment*, *176*(1), 235-242. <https://doi.org/10.1007/s10549-019-05225-9>

Davidson, P. M., Jiwa, M., Digiacomo, M. L., McGrath, S. J., Newton, P. J., Durey, A. J., Bessarab, D. C., & Thompson, S. C. (2013). The experience of lung cancer in Aboriginal and Torres Strait Islander peoples and what it means for policy, service planning and delivery. *Aust Health Rev*, *37*(1), 70-78. <https://doi.org/10.1071/AH10955>

De Plaza, M. A. P., Gebremichael, L., Brown, S., Wu, C.-J., Clark, R. A., McBride, K., Hines, S., Pearson, O., & Morey, K. (2023). Health system enablers and barriers to continuity of care for first nations peoples living with chronic disease. *International journal of integrated care*, *23*(4), 17.

de Witt, A., Cunningham, F. C., Bailie, R., Bernardes, C. M., Matthews, V., Arley, B., Meiklejohn, J. A., Garvey, G., Adams, J., Martin, J. H., Walpole, E. T., Williamson, D., & Valery, P. C. (2017). Identification of Australian Aboriginal and Torres Strait Islander Cancer Patients in the Primary Health Care Setting. *Front Public Health*, *5*, 199. <https://doi.org/10.3389/fpubh.2017.00199>

de Witt, A., Cunningham, F. C., Bailie, R., Percival, N., Adams, J., & Valery, P. C. (2018). "It's Just Presence," the Contributions of Aboriginal and Torres Strait Islander Health Professionals in Cancer Care in Queensland [Original Research]. *Front Public Health*, *6*(344), 344. <https://doi.org/10.3389/fpubh.2018.00344>

de Witt, A., Matthews, V., Bailie, R., Garvey, G., Valery, P. C., Adams, J., Martin, J. H., & Cunningham, F. C. (2020). Communication, Collaboration and Care Coordination: The Three-Point Guide to Cancer Care Provision for Aboriginal and Torres Strait Islander Australians. *International Journal of Integrated Care*, *20*(2), 10. <https://doi.org/10.5334/ijic.5456>

de Witt, A., Matthews, V., Bailie, R., Valery, P. C., Adams, J., Garvey, G., Martin, J. H., & Cunningham, F. C. (2021). Aboriginal and Torres Strait Islander patients' cancer care pathways in Queensland: Insights from health professionals. *Health Promot J Austr*. <https://doi.org/10.1002/hpja.556>

Diaz, A., Vo, B., Baade, P. D., Matthews, V., Nattabi, B., Bailie, J., Whop, L. J., Bailie, R., & Garvey, G. (2019). Service Level Factors Associated with Cervical Screening in Aboriginal and Torres Strait Islander Primary Health Care Centres in Australia. *Int J Environ Res Public Health*, *16*(19), 3630. <https://doi.org/10.3390/ijerph16193630>

Gall, A., Anderson, K., Adams, J., Matthews, V., & Garvey, G. (2019). An exploration of healthcare providers' experiences and perspectives of Traditional and complementary medicine usage and disclosure by Indigenous cancer patients. *BMC Complement Altern Med*, *19*(1), 259. <https://doi.org/10.1186/s12906-019-2665-7>

Gall, A., Anderson, K., Diaz, A., Matthews, V., Adams, J., Taylor, T., & Garvey, G. (2019). Exploring traditional and complementary medicine use by Indigenous Australian women undergoing gynaecological cancer investigations [Article]. *Complement Ther Clin Pract*, *36*, 88-93. <https://doi.org/10.1016/j.ctcp.2019.06.005>

Gall, A., Leske, S., Adams, J., Matthews, V., Anderson, K., Lawler, S., & Garvey, G. (2018). Traditional and Complementary Medicine Use Among Indigenous Cancer Patients in Australia, Canada, New Zealand, and the United States: A Systematic Review. *Integr Cancer Ther*, *17*(3), 568-581. <https://doi.org/10.1177/1534735418775821>

Haigh, M., Shahid, S., O'Connor, K., & Thompson, S. C. (2016). Talking about the not talked about: use of, and reactions to, a DVD promoting bowel cancer screening to Aboriginal people. *Aust N Z J Public Health*, *40*(6), 548-552. <https://doi.org/10.1111/1753-6405.12565>

Lethborg, C., Halatanu, F., Mason, T., Posenelli, S., Cleak, H., & Braddy, L. (2022). Culturally informed, codesigned, supportive care for Aboriginal and Torres Strait Islander people with cancer and their families. *Australian Social Work*, *75*(2), 165-179.

Lyford, M., Haigh, M., Baxi, S., Cheetham, S., Shahid, S., & Thompson, S. (2018). An Exploration of Underrepresentation of Aboriginal Cancer Patients Attending a Regional Radiotherapy Service in Western Australia [Journal Article]. *Int J Environ Res Public Health*, *15*(2), 337. <https://doi.org/10.3390/ijerph15020337>

Martini, A., Javanparast, S., Ward, P. R., Baratiny, G., Gill, T., Cole, S., Tsourtos, G., Aylward, P., Jiwa, M., Misan, G., Wilson, C., & Young, G. P. (2011). Colorectal cancer screening in rural and remote areas: analysis of the National Bowel Cancer Screening Program data for South Australia. *Rural Remote Health*, *11*(2), 1648. <https://www.ncbi.nlm.nih.gov/pubmed/21585228>

McLean, K., Darcey, E., Cadby, G., Lund, H., Pilkington, L., Redfern, A., Thompson, S., Saunders, C., Wylie, E., & Stone, J. (2019). The distribution and determinants of mammographic density measures in Western Australian aboriginal women. *Breast Cancer Research*, *21*(33). <https://doi.org/10.1186/s13058-019-1113-4>

Meiklejohn, J. A., Arley, B., Bailie, R., Adams, J., Garvey, G., Martin, J. H., Walpole, E. T., & Valery, P. C. (2018). Community-identified recommendations to enhance cancer survivorship for Aboriginal and Torres Strait Islander people. *Aust J Prim Health*, *24*(3), 233-240. <https://doi.org/10.1071/PY17127>

Meiklejohn, J. A., Garvey, G., Bailie, R., Walpole, E., Adams, J., Williamson, D., Martin, J., Bernardes, C. M., Arley, B., Marcusson, B., & Valery, P. C. (2017). Follow-up cancer care: perspectives of Aboriginal and Torres Strait Islander cancer survivors. *Support Care Cancer*, *25*(5), 1597-1605. <https://doi.org/10.1007/s00520-016-3563-x>

Olver, I., Gunn, K. M., Chong, A., Knott, V., Spronk, K., Cominos, N., & Cunningham, J. (2021). Communicating cancer and its treatment to Australian Aboriginal and Torres Strait Islander patients with cancer: a qualitative study. *Support Care Cancer*, *30*(1), 431-438. <https://doi.org/10.1007/s00520-021-06430-3>

Panozzo, S., Bryan, T., Mason, T., Garvey, G., Lethborg, C., Boughey, M., & Philip, J. A. (2023). Bridging cultures in palliative care: a qualitative study of the care of Indigenous Australians with advanced illness. *Palliative Medicine*, *37*(4), 498-507.

Pilkington, L., Haigh, M. M., Durey, A., Katzenellenbogen, J. M., & Thompson, S. C. (2017). Perspectives of Aboriginal women on participation in mammographic screening: a step towards improving services [journal article]. *BMC Public Health*, *17*(1), 697. <https://doi.org/10.1186/s12889-017-4701-1>

Ristevski, E., Thompson, S., Kingaby, S., Nightingale, C., & Iddawela, M. (2020). Understanding Aboriginal Peoples' Cultural and Family Connections Can Help Inform the Development of Culturally Appropriate Cancer Survivorship Models of Care. *JCO Glob Oncol*, *6*, 124-132. <https://doi.org/10.1200/JGO.19.00109>

Shahid, S., Bessarab, D., van Schaik, K. D., Aoun, S. M., & Thompson, S. C. (2013). Improving palliative care outcomes for Aboriginal Australians: service providers' perspectives. *BMC Palliat Care*, *12*(1), 26. <https://doi.org/10.1186/1472-684X-12-26>

Shahid, S., Bleam, R., Bessarab, D., & Thompson, S. C. (2010). "If you don't believe it, it won't help you": use of bush medicine in treating cancer among Aboriginal people in Western Australia. *Journal of Ethnobiology and Ethnomedicine*, *6*. <https://doi.org/Artn> 18

10.1186/1746-4269-6-18

Shahid, S., Durey, A., Bessarab, D., Aoun, S. M., & Thompson, S. C. (2013). Identifying barriers and improving communication between cancer service providers and Aboriginal patients and their families: the perspective of service providers. *BMC Health Serv Res*, *13*(1), 460. <https://doi.org/10.1186/1472-6963-13-460>

Shahid, S., Ekberg, S., Holloway, M., Jacka, C., Yates, P., Garvey, G., & Thompson, S. C. (2018). Experiential learning to increase palliative care competence among the Indigenous workforce: an Australian experience. *BMJ Supportive and Palliative Care*, *Online Open Access*, 1-6. <https://doi.org/10.1136/bmjspcare-2016-001296>

Shahid, S., Finn, L., Bessarab, D., & Thompson, S. C. (2011). 'Nowhere to room ... nobody told them': logistical and cultural impediments to Aboriginal peoples' participation in cancer treatment. *Aust Health Rev*, *35*(2), 235-241. <https://doi.org/10.1071/AH09835>

Shahid, S., Taylor, E. V., Cheetham, S., Woods, J. A., Aoun, S. M., & Thompson, S. C. (2018). Key features of palliative care service delivery to Indigenous peoples in Australia, New Zealand, Canada and the United States: a comprehensive review [Research Journal Article ]. *BMC Palliat Care*, *17*(1), 72. <https://doi.org/10.1186/s12904-018-0325-1>

Shahid, S., Teng, T. H., Bessarab, D., Aoun, S., Baxi, S., & Thompson, S. C. (2016). Factors contributing to delayed diagnosis of cancer among Aboriginal people in Australia: a qualitative study. *BMJ Open*, *6*(6), e010909. <https://doi.org/10.1136/bmjopen-2015-010909>

Shepherdson, M., Leemaqz, S., Singh, G., Ryder, C., Ullah, S., Canuto, K., Young, J. P., Price, T. J., McKinnon, R. A., & Pandol, S. J. (2022). Young-Onset Gastrointestinal Adenocarcinoma Incidence and Survival Trends in the Northern Territory, Australia, with Emphasis on Indigenous Peoples. *Cancers*, *14*(12), 2870.

Smith, T. (2012). A long way from home: Access to cancer care for rural Australians. *Radiography*, *18*(1), 38-42. <https://doi.org/10.1016/j.radi.2011.10.041>

Spelten, E. R., MacDermott, S., Morgan, S., Mitchell, L., & van Vuuren, J. (2021). Palliative Care in Rural Aboriginal Communities: Conversations Around Experiences and Needs. *J Hosp Palliat Nurs*, *23*(6), 579-583. <https://doi.org/10.1097/NJH.0000000000000801>

Taylor, E. V., Haigh, M. M., Shahid, S., Garvey, G., Cunningham, J., Holloway, M., & Thompson, S. C. (2018). Australian cancer services: a survey of providers’ efforts to meet the needs of Indigenous patients. *Aust N Z J Public Health*, *0*(0), 1-6. <https://doi.org/doi:10.1111/1753-6405.12843>

Taylor, E. V., Haigh, M. M., Shahid, S., Garvey, G., Cunningham, J., & Thompson, S. C. (2018). Cancer Services and Their Initiatives to Improve the Care of Indigenous Australians. *Int J Environ Res Public Health*, *15*(4). <https://doi.org/10.3390/ijerph15040717>

Taylor, E. V., Lyford, M., Holloway, M., Parsons, L., Mason, T., Sabesan, S., & Thompson, S. C. (2021). "The support has been brilliant": experiences of Aboriginal and Torres Strait Islander patients attending two high performing cancer services. *BMC Health Serv Res*, *21*(1), 493. <https://doi.org/10.1186/s12913-021-06535-9>

Taylor, E. V., Lyford, M., Parsons, L., Holloway, M., Gough, K., Sabesan, S., & Thompson, S. C. (2022). Putting policy into practice: how three cancer services perform against indigenous health and cancer frameworks. *International journal of environmental research and public health*, *19*(2), 633.

Taylor, E. V., Lyford, M., Parsons, L., Mason, T., Sabesan, S., & Thompson, S. C. (2020). "We're very much part of the team here": A culture of respect for Indigenous health workforce transforms Indigenous health care. *PLoS One*, *15*(9), e0239207. <https://doi.org/10.1371/journal.pone.0239207>

Taylor, E. V., Thackrah, R. D., & Thompson, S. C. (2022). Improving Access to Cancer Treatment Services in Australia’s Northern Territory—History and Progress. *International journal of environmental research and public health*, *19*(13), 7705.

Thackrah, R. D., Papertalk, L. P., Taylor, K., Taylor, E. V., Greville, H., Pilkington, L. G., & Thompson, S. C. (2022). Perspectives of aboriginal people affected by cancer on the need for an aboriginal navigator in cancer treatment and support: a qualitative study. Healthcare,

Thompson, S., Lyford, M., Papertalk, L., & Holloway, M. (2019). Passing on wisdom: exploring the end-of-life wishes of Aboriginal people from the Midwest of Western Australia [Journal Article]. *Rural Remote Health*, *19*(4), 5444. <https://doi.org/10.22605/RRH5444>

Thompson, S. C., Shahid, S., Bessarab, D., Durey, A., & Davidson, P. M. (2011). Not just bricks and mortar: planning hospital cancer services for Aboriginal people. *BMC Res Notes*, *4*, 62. <https://doi.org/10.1186/1756-0500-4-62>

Thompson, S. C., Shahid, S., DiGiacomo, M., Pilkington, L., & Davidson, P. M. (2014). Making progress: the role of cancer councils in Australia in indigenous cancer control. *BMC Public Health*, *14*(1), 347. <https://doi.org/10.1186/1471-2458-14-347>

Tranberg, R., Alexander, S., Hatcher, D., Mackey, S., Shahid, S., Holden, L., & Kwok, C. (2016). Factors influencing cancer treatment decision-making by indigenous peoples: a systematic review. *Psychooncology*, *25*(2), 131-141. <https://doi.org/10.1002/pon.3900>

Valery, P. C., Bernardes, C. M., de Witt, A., Martin, J., Walpole, E., Garvey, G., Williamson, D., Meiklejohn, J., Hartel, G., Ratnasekera, I. U., & Bailie, R. (2020a). Are general practitioners getting the information they need from hospitals and specialists to provide quality cancer care for Indigenous Australians? *Intern Med J*, *50*(1), 38-47. <https://doi.org/10.1111/imj.14356>

Valery, P. C., Bernardes, C. M., de Witt, A., Martin, J., Walpole, E., Garvey, G., Williamson, D., Meiklejohn, J., Hartel, G., Ratnasekera, I. U., & Bailie, R. (2020b). Patterns of primary health care service use of Indigenous Australians diagnosed with cancer [journal article]. *Support Care Cancer*, *28*(1), 317-327. <https://doi.org/10.1007/s00520-019-04821-1>

van Schaik, K. D., & Thompson, S. C. (2012). Indigenous beliefs about biomedical and bush medicine treatment efficacy for indigenous cancer patients: a review of the literature. *Intern Med J*, *42*(2), 184-191. <https://doi.org/10.1111/j.1445-5994.2011.02598.x>

Ward, P. R., Javanparast, S., Matt, M. A., Martini, A., Tsourtos, G., Cole, S., Gill, T., Aylward, P., Baratiny, G., Jiwa, M., Misan, G., Wilson, C., & Young, G. (2011). Equity of colorectal cancer screening: cross-sectional analysis of National Bowel Cancer Screening Program data for South Australia. *Aust N Z J Public Health*, *35*(1), 61-65. <https://doi.org/10.1111/j.1753-6405.2010.00637.x>

Woods, J. A., & Johnson, C. E. (2018). Delay in commencement of palliative care service episodes provided to Indigenous and non-Indigenous patients: cross-sectional analysis of an Australian multi-jurisdictional dataset. *BMC Palliat Care*, *17*(1), 130. <https://doi.org/10.1186/s12904-018-0380-7>

Woods, J. A., Johnson, C. E., Allingham, S. F., Ngo, H. T., Katzenellenbogen, J. M., & Thompson, S. C. (2021). Collaborative data familiarisation and quality assessment: Reflections from use of a national dataset to investigate palliative care for Indigenous Australians. *Health Inf Manag*, *50*(1-2), 64-75. <https://doi.org/10.1177/1833358320908957>

Woods, J. A., Johnson, C. E., Ngo, H. T., Katzenellenbogen, J. M., Murray, K., & Thompson, S. C. (2020). Symptom-Related Distress among Indigenous Australians in Specialist End-of-Life Care: Findings from the Multi-Jurisdictional Palliative Care Outcomes Collaboration Data. *Int J Environ Res Public Health*, *17*(9), 3131. <https://doi.org/10.3390/ijerph17093131>

Woods, J. A., Katzenellenbogen, J. M., Murray, K., Johnson, C. E., & Thompson, S. C. (2021). Occurrence and timely management of problems requiring prompt intervention among Indigenous compared with non-Indigenous Australian palliative care patients: a multijurisdictional cohort study. *BMJ Open*, *11*(3), e042268. <https://doi.org/10.1136/bmjopen-2020-042268>

Woods, J. A., Newton, J. C., Thompson, S. C., Malacova, E., Ngo, H. T., Katzenellenbogen, J. M., Murray, K., Shahid, S., & Johnson, C. E. (2019). Indigenous compared with non-Indigenous Australian patients at entry to specialist palliative care: Cross-sectional findings from a multi-jurisdictional dataset. *PLoS One*, *14*(5), e0215403. <https://doi.org/10.1371/journal.pone.0215403>
