# Supplementary figures and images for: University Departments of Rural Health and cancer research in Aboriginal and Torres Strait Islander populations
Source: Lancet Reg Health West Pac. 2025 Jun 29;61:101621. doi: 10.1016/j.lanwpc.2025.101621 (PMC12414349; doi:10.1016/j.lanwpc.2025.101621)

**Supplementary file 2.**

Number of UDRH publications relating to cancer between 2010-2023

**
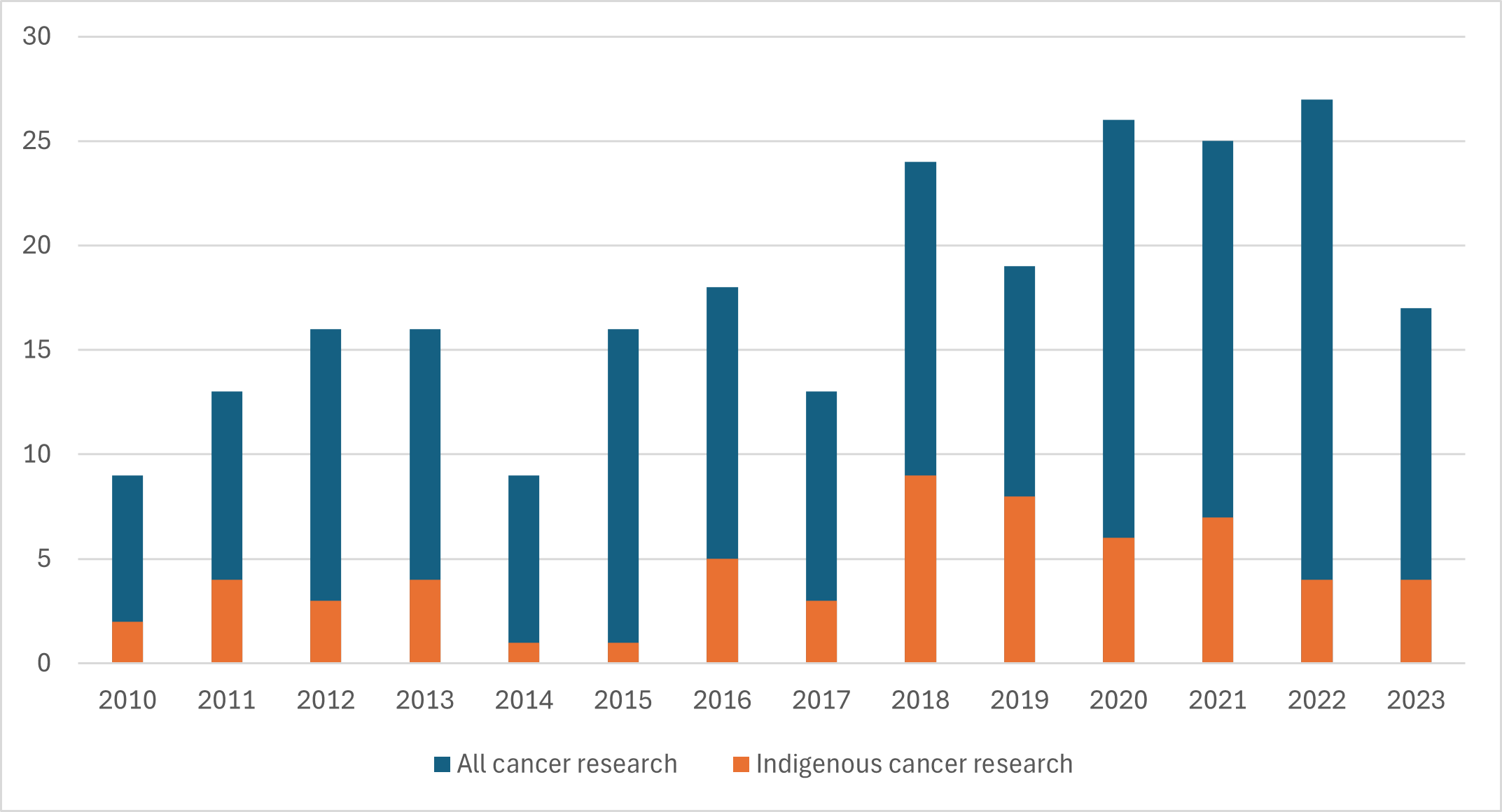
**

Supplement: Supplementary file S2 [file mmc2.docx]
